# P16^INK4A^ drives RB1 degradation by UTP14A-catalyzed K810 ubiquitination

**DOI:** 10.1016/j.isci.2024.110882

**Published:** 2024-09-03

**Authors:** Wenjie Weng, Baozhen Zhang, Dajun Deng

**Affiliations:** 1 Key Laboratory of Carcinogenesis and Translational Research (MOE/Beijing) Division of Etiology, Peking University Cancer Hospital and Institute, Beijing 100142, China

**Keywords:** Cellular physiology, Molecular physiology, Molecular interaction, Functional aspects of cell biology

## Abstract

P16^INK4A^ expression is inversely associated with RB1 expression in cancer cells, and P16^INK4A^ inhibits CDK4-catalyzed RB1 phosphorylation. How P16^INK4A^ and RB1 coordinately express and regulate the cell cycle remains to be studied. In the present study, we found that P16^INK4A^ upregulated the E3 ligase UTP14A, which led to the ubiquitination of RB1 at K810 and RB1 degradation. P16^INK4A^ loss consistently disrupted the UTP14A-mediated degradation of RB1 and caused RB1 accumulation. Functionally, P16^INK4A^ loss inhibited RB1 ubiquitination in a cell cycle progression-independent fashion and inhibited proteome-scale ubiquitination in a cell cycle progression-dependent manner. Our findings indicate that there is a negative feedback loop between P16^INK4A^ and RB1 expression and that disruption of this loop may partially rescue the biological outcomes of P16^INK4A^ loss. We also revealed a hitherto unknown function for *P16*^*INK4A*^ in regulating proteome-scale ubiquitination by inhibiting cell proliferation, which may be useful for the development of anticancer drugs.

## Highlights

• We discovered that P16^INK4A^ drives RB1 degradation through ubiquitination at K810
• We found that the E3 UTP14A is essential for P16^INK4A^-mediated RB1 degradation
• We revealed that P16^INK4A^ reduces UTP14A degradation
• Loss of P16 enhances RB1 accumulation in a cell cycle-independent fashion

## Graphical abstract

**Figure:**
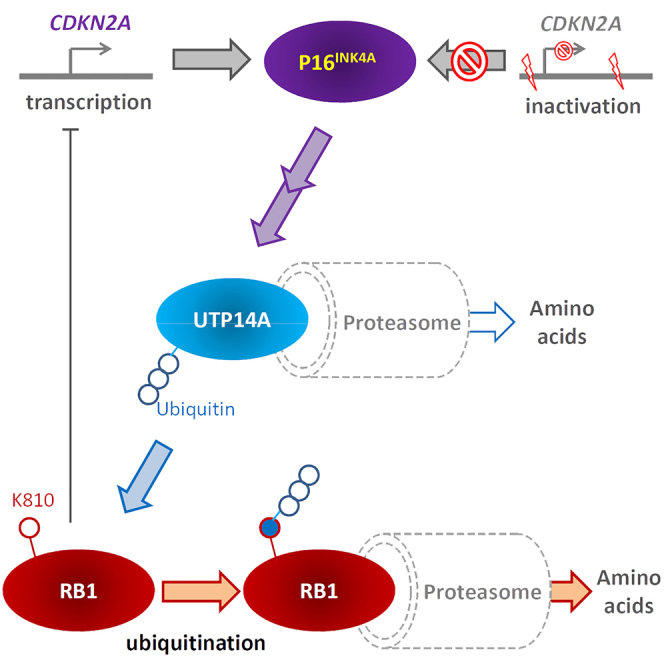

## Introduction

Alterations in regulators of the cell cycle are among the hallmarks of cancer cells. Among them, the tumor suppressor genes *CDKN2A* and *RB1* are frequently inactivated by copy number deletions or point mutations, whereas the oncogenes *CDK4*, *CDK2*, *CCND1*, and *CCNE1* are recurrently amplified in the genomes of some human cancers.^1^ The *CDKN2A*-encoded protein P16^INK4A^ (P16) inhibits CDK4 kinase activity, RB1 phosphorylation, and the G1‒S phase transition in the cell cycle.^2^^,^^3^ Currently, CDK4 is a useful therapeutic target. Several small chemical CDK4 inhibitors, such as palbociclib, have been clinically approved for the treatment of patients with HER2-negative and ER-positive breast cancers.^4^ Although P16 is an endogenous CDK4 inhibitor, only a few primary cancers with *P16* inactivation may be sensitive to treatment with CDK4 inhibitors.^5^^,^^6^

In general, inactivation of one cell cycle-related gene often results in compensatory upregulation of other cell cycle-related genes.^7^^,^^8^^,^^9^^,^^10^^,^^11^ For example, P16 downregulation leads to P14^ARF^ and P15^INK4B^ upregulation in mouse fibroblasts,^10^ and RB1 dysfunction caused by HPV-E6 results in P16 overexpression in human cells.^11^ The complex regulatory responses of cancer cells to *CDKN2A* inactivation may account for the development of resistance in cancer cells to CDK4 inhibitors. Somatic copy number deletion of the *CDKN2A* gene is a transformed cell-specific event, which prompted scientists to study whether disruption of the *CDKN2A* deletion-related feedback regulatory loop may overcome the resistance of *P16*-inactivated cancer cells to CDK4 inhibitors. Although many efforts have been made,^12^^,^^13^^,^^14^^,^^15^ the regulatory networks between cell cycle-related genes remain to be studied.

RB1 is essential for CDK4 inhibitors to inhibit cancer cell proliferation. Although RB1 expression is consistently and inversely associated with P16 expression, whether P16 affects RB1 stability is unknown. Our present study illustrates that the P16 protein has an unknown function. P16 drives the feedforward degradation of RB1 through UTP14A-catalyzed K810 ubiquitination, which results in the formation of a negative feedback regulatory loop between P16 and RB1 expression in human cells. Loss of P16 disrupts the loop and consequently stabilizes the RB1 protein.

## Results

### P16 promotes posttranscriptional downregulation of the RB1 protein

By mining RNA sequencing (RNA-seq) and proteomic datasets from the Cancer Cell Line Encyclopedia (CCLE),^16^^,^^17^ we found that there was an inverse relationship between the expression levels of the *CDKN2A* and *RB1* genes, especially at the protein level, among human cancer cell lines (*N* = 364, R = −0.38, and *p* = 8.74e-14; Figures S1A and S1B). Our western blot results confirmed the bioinformatics analysis results (Figures S1C and S1D). The average abundance of the RB1 protein was greater in cancer cell lines without P16 expression than in those with P16 expression.

**Figure 1:**
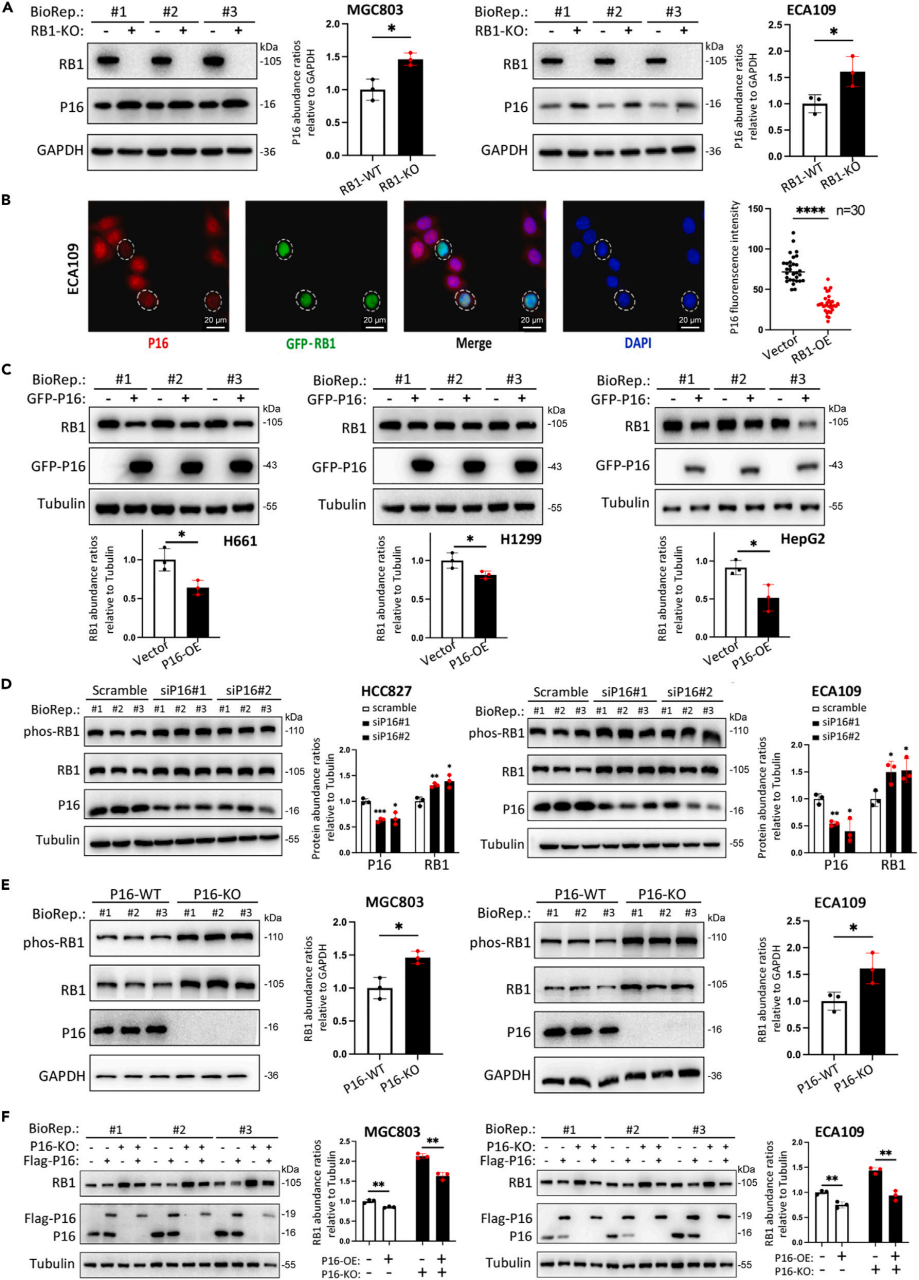
Reciprocal feedback regulation between RB1 and CDKN2A/P16 expression (A) Impact of *RB1* knockout (RB1-KO) on the abundance of the P16 protein in MGC803 and ECA109 cancer cells. (B) Effect of GFP-RB1 overexpression on P16 abundance in individual ECA109 cells. The fluorescence intensity was observed with a confocal microscope. (C) Impact of transient GFP-P16 overexpression on the abundance of the RB1 protein in H661, H1299, and HepG2 cells. (D) Impact of siRNA-mediated knockdown of *P16* expression (siP16#1 and #2) on the abundance of phosphorylated RB1 (phos-RB1) and total RB1 in HCC827 and ECA109 cells. (E) Impact of *P16* knockout (P16-KO) on the abundance of phos-RB1 and total RB1 in MGC803 and ECA109 cells. (F) Effect of restoring *P16* expression on P16-KO-induced RB1 upregulation in MGC803 and ECA109 cells. (A and C–F) The density of target protein bands in the western blot analyses was normalized to that of the GAPDH or tubulin reference and is displayed on the right side as the mean with the standard deviation (SD) for 3 biological replicates (BioRep). ∗/∗∗/∗∗∗/∗∗∗∗: *p* < 0.05/0.01/0.001/0.0001 according to unpaired Student’s t test.

It has been reported that a lack of RB1 function may induce feedback upregulation of P16.^7^^,^^8^^,^^9^ Consistently, we found that genetic *RB1* knockout (RB1-KO) via CRISPR-Cas9 indeed increased the abundance of P16 in MGC803 and ECA109 cancer cells (Figures 1A and S2A), whereas transient GFP-RB1 overexpression (RB1-OE) significantly decreased the abundance of P16 (Figure 1B). Conversely, transient P16 overexpression (P16-OE) markedly decreased the abundance of RB1 in H661, H1299, and HepG2 cells (Figure 1C). Both small interfering RNAs (siRNA)-mediated knockdown of *P16* expression (siP16) and KO of *CDKN2A* exon 1α (an essential exon for P16 synthesis; P16-KO; Figure S2B) significantly increased the abundance of both phosphorylated RB1 at S807/S811 (phos-RB1) and total RB1 (with and without phosphorylation; Figures 1D and 1E). Restoration of P16 expression mitigated the impact of P16 KO on the abundance of RB1 (Figure 1F). These results demonstrate that there is a reciprocal regulatory feedback loop between RB1 and P16 expression. Loss of P16 function induces compensatory RB1 upregulation and vice versa.

### P16 promotes the degradation of RB1 through ubiquitination

We found that P16-OE, siP16, and P16-KO did not change the level of *RB1* mRNA (Figures S3A and S3B), whereas RB1-KO increased the *P16* mRNA level (Figure S3C). This finding suggests a posttranscriptional mechanism by which P16 decreases RB1 expression.

**Figure 2:**
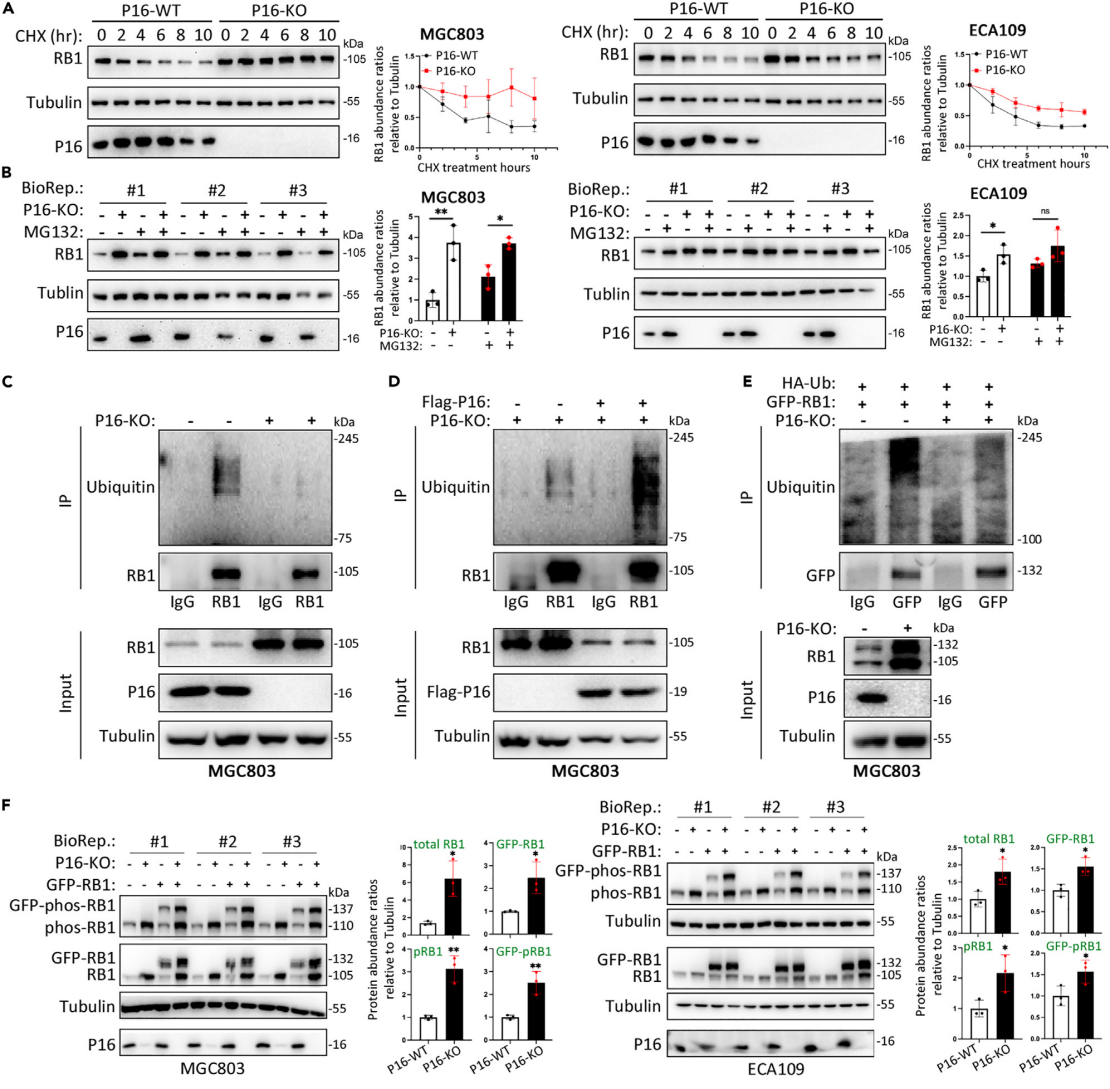
Effect of P16 on RB1 degradation via ubiquitination (A) Status of RB1 stability in P16-WT and P16-KO cells treated with or without cycloheximide (CHX; final concentration, 50 μg/mL) at various time points, as determined by western blotting. (B) Status of RB1 stability in P16-WT and P16-KO cells treated with or without MG132 (25 μM) for 4 h. (C) Impact of P16-KO on the ubiquitination of endogenous RB1 in cells treated with the proteasome inhibitor MG132. (D) Impact of the restoration of P16 expression on the P16-KO-repressed ubiquitination of endogenous RB1. (E) Impact of P16-KO on the ubiquitination of overexpressed GFP-RB1 in MGC803 cells transfected with HA-Ub plasmids. (F) Impact of P16-KO on the abundance of various RB1 forms, including phosphorylated and total endogenous RB1 and exogenous GFP-RB1. (A, B, and F) Three biological replicates (BioRep).

When novel protein synthesis was blocked by cycloheximide (CHX) treatment, the abundance of RB1 slightly, if any, decreased in P16-KO cells, whereas it greatly decreased in *P16* wild-type (WT) (P16-WT) control cells (Figure 2A). Treatment with the proteasome inhibitor MG132 mitigated the P16-KO-mediated increase in the abundance of RB1 (Figure 2B). These phenomena revealed that P16 loss may increase the stability of RB1 through the inhibition of the ubiquitin-proteasome pathway.

Immunoprecipitation (IP) analysis revealed that the abundance of ubiquitinated RB1 was markedly decreased in P16-KO cells (Figure 2C) and that restoring *P16* expression with the FLAG-P16 expression vector greatly increased the abundance of ubiquitinated RB1 in P16-KO cells (Figure 2D). Similarly, exogenous GFP-RB1 was much less ubiquitinated by HA-labeled ubiquitin (HA-Ub) in P16-KO cells than in P16-WT cells (Figure 2E). P16-KO not only increased the abundance of phosphorylated and total endogenous RB1 but also increased that of exogenous GFP-RB1 (Figure 2F). These results support the aforementioned hypothesis that *P16* inactivation increases the stability of RB1 through the inhibition of its ubiquitination.

### K810 ubiquitination contributed to the P16-mediated degradation of RB1

**Figure 3:**
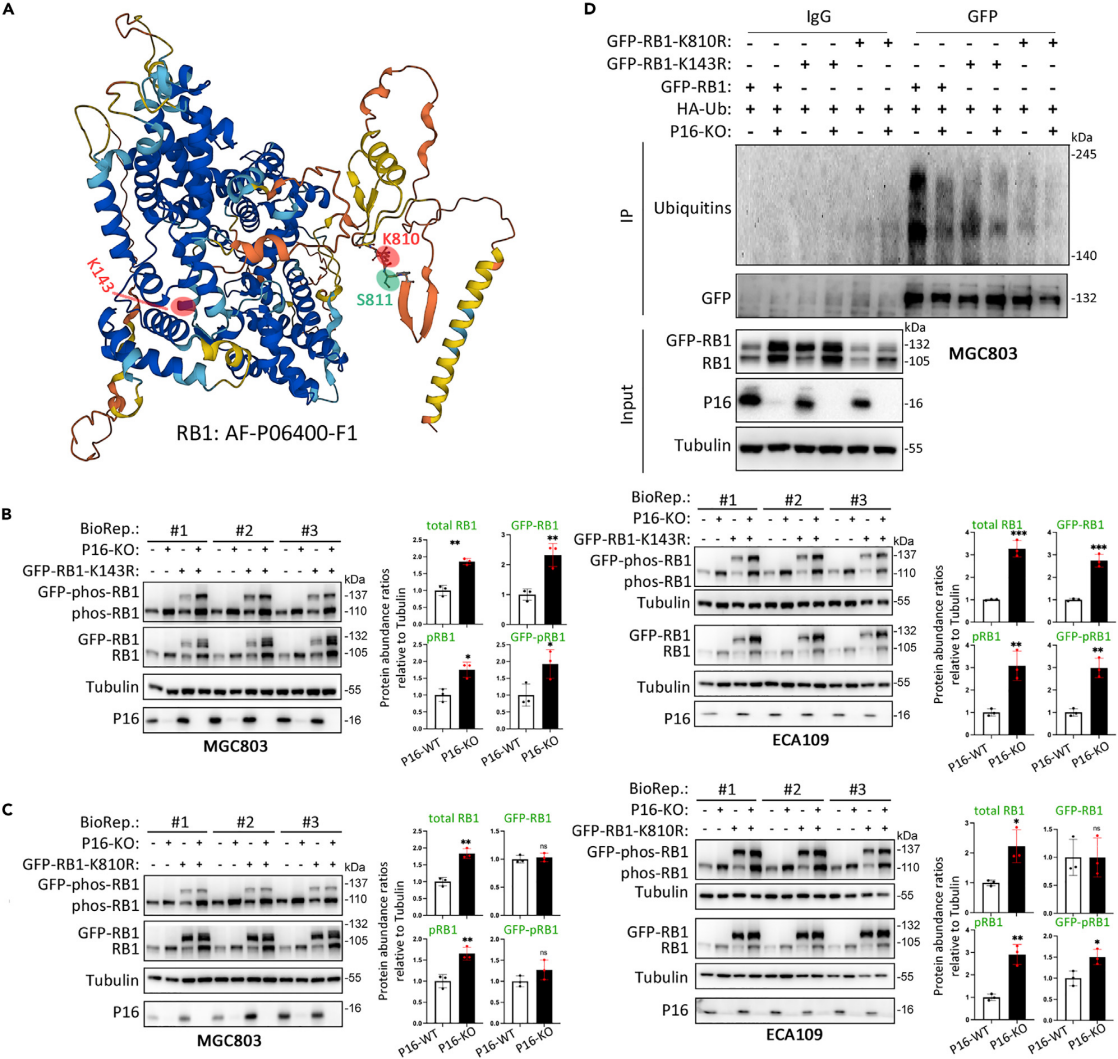
Characterization of P16-KO-related ubiquitination sites within RB1 (A) Graphical structure of RB1 predicted by AlphaFold 2. Two known ubiquitination sites (K143 and K810) and one phosphorylation site, S811, are marked. (B and C) Effects of the K143R and K810R mutations on the abundance of various RB1 forms, including total RB1 and phos-RB1, and overexpressed RB1 in P16-KO ECA109 and MGC803 cells. (D) Impact of the K143R and K810R mutations on the P16-KO-mediated repression of endogenous and exogenous RB1 ubiquitination in MGC803 cells treated with the proteasome inhibitor MG132 and transfected with the HA-Ub vector. (B and C) Three biological replicates (BioRep).

Although the degradation of RB1 by the ubiquitination-proteasome pathway has been reported and a number of ubiquitination sites within RB1 have been characterized by mass spectrometry (MS), the exact contribution of ubiquitination at these sites to RB1 degradation is unclear.^18^^,^^19^^,^^20^^,^^21^^,^^22^^,^^23^^,^^24^^,^^25^ According to several proteome-wide quantitative surveys of *in vivo* ubiquitination,^26^^,^^27^^,^^28^^,^^29^ there are two recurrent ubiquitination sites within RB1 at the 143rd and 810th lysines (K143 and K810; Figure 3A). K810 is also methylated by SET7/9 and SMYD2 and has multiple functions.^30^^,^^31^^,^^32^^,^^33^^,^^34^ Thus, we constructed two RB1 expression mutants through the replacement of lysine with arginine (K143R or K810R) to abolish the ubiquitination potential at these sites. Notably, P16-KO still increased the abundance of the exogenous RB1-K143R mutant and endogenous RB1 (Figure 3B). However, P16 KO did not affect the abundance of the RB1-K810R mutant (Figure 3C). IP analysis also revealed that the level of ubiquitinated RB1-K810R was greatly decreased in both P16-KO and P16-WT cells, whereas that of ubiquitinated RB1-K143R was only slightly decreased (Figure 3D). Collectively, these experimental results strongly support that the absence of P16 inhibits the K810 ubiquitination of RB1 and subsequently increases RB1 stability.

### UTP14A contributes to the P16-mediated ubiquitination of RB1

**Figure 4:**
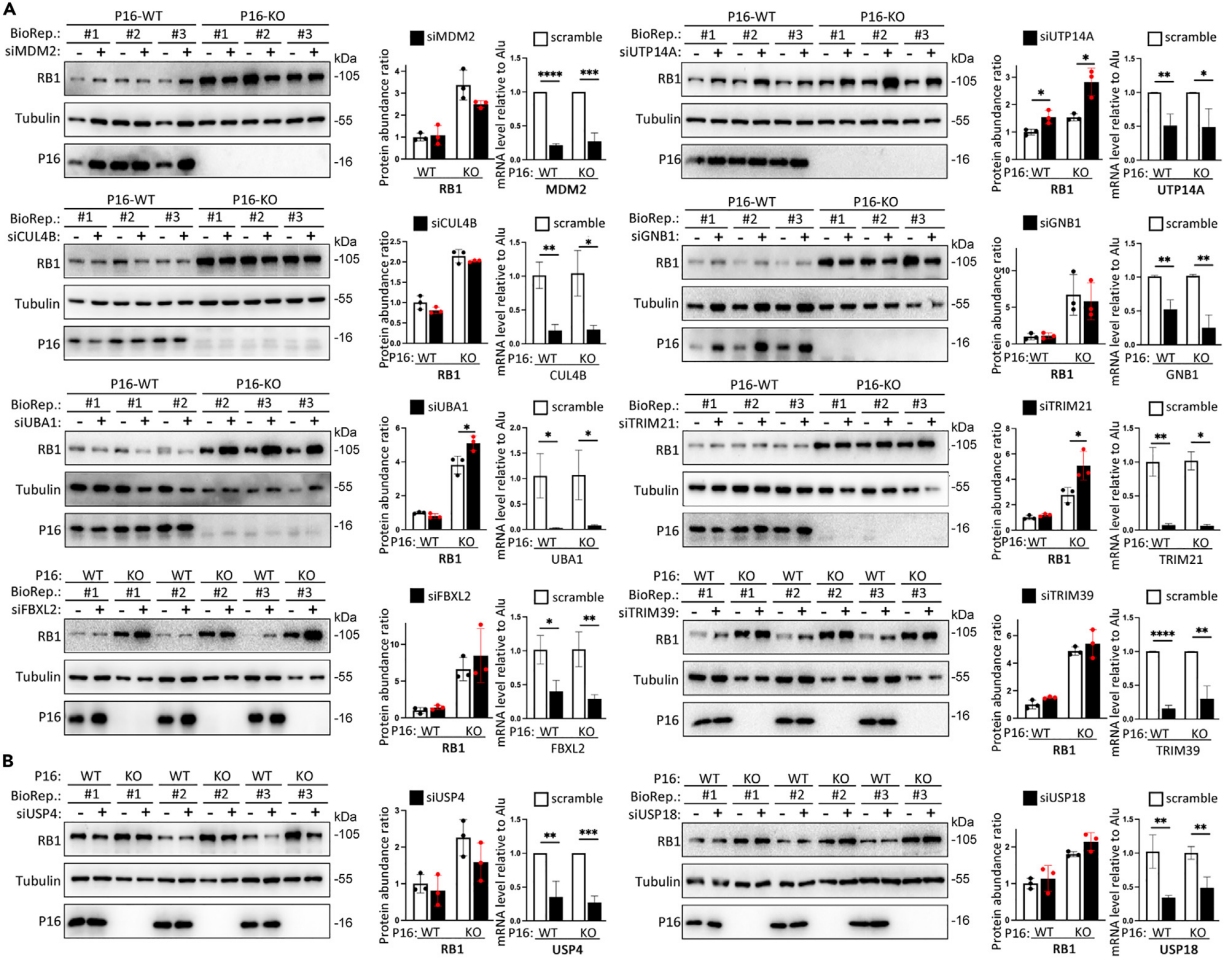
RB1 ubiquitination-related enzymes in MGC803 gastric cancer cells were screened with a small interfering RNA (siRNA) array (A) Effects of siRNA-mediated knockdown of seven E3 ligases (MDM2, UTP14A, CUL4B, GNB1, FBXL2, TRIM21, and TRIM39) and E1 ligase (UBA1) on the abundance of RB1 in P16-WT and P16-KO cells, as determined by western blotting. (B) Effect of siRNA-mediated knockdown of two deubiquitinases (USP4 and USP18) on the abundance of RB1 in P16-WT and P16-KO cells. The density of the band for RB1 was normalized to that for tubulin and is listed in the middle charts. The knockdown status of target gene expression with siRNAs was detected via RT-qPCR and is presented on the right. The average and standard deviation (SD) values for the abundance of RB1 in three biological replicates (BioRep) were calculated.

Both decreased ubiquitination activity and increased deubiquitination activity may lead to the accumulation of target proteins. There are several known RB1 ubiquitinases (E3 ligases, including SKP2,^18^ MDM2,^19^^,^^20^ TRIM71,^21^ E6AP/UBE3A,^22^ UTP14A,^24^ and NRBE3^25^), deubiquitinases (USP4 and USP7), and a number of predicted ubiquitinases (Data S1).^26^^,^^27^^,^^28^^,^^29^ Accordingly, we used a set of siRNAs to knock down the expression of 7 ubiquitin E3 ligases (CUL4B, GNB1, FBXL2, MDM2, TRIM21, TRIM39, and UTP14A), 1 ubiquitin-activating enzyme E1 (UBA1), and 2 deubiquitinases (USP4 and USP18) to screen for ubiquitination modulators that mediate the P16-mediated degradation of RB1 (Figures 4A and 4B). We found that transient knockdown of *UTP14A* expression (siUTP14A) mostly increased RB1 levels in P16-KO MGC803 cells, whereas it only slightly increased RB1 levels in P16-WT cells. Moreover, knockdown of E1 *UBA1* expression (siUBA1) markedly increased the abundance of RB1 in P16-KO cells. In contrast, knockdown of *MDM2* expression (siMDM2) decreased the abundance of RB1 in P16-KO cells. In addition, MS analysis revealed that UTP14A (Q9BVJ6) was the only ubiquitin E3 ligase that differentially bound to RB1 in P16-WT cells (Data S2). Therefore, we further studied the contribution of UTP14A to the P16-KO-mediated inhibition of RB1 ubiquitination and degradation in detail.

**Figure 5:**
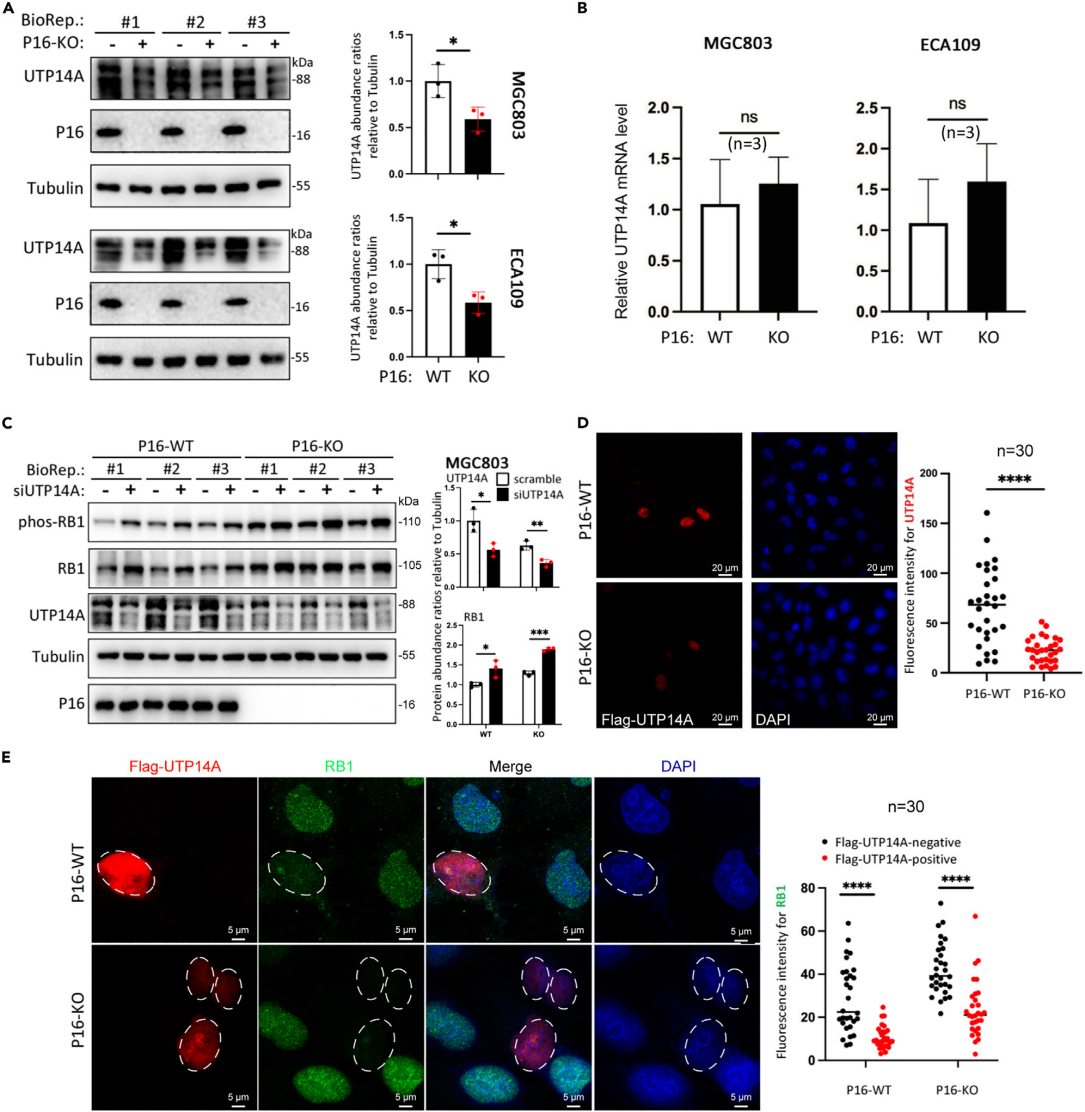
Effect of changes in UTP14A expression on RB1 degradation induced by P16 (A and B) Changes in the expression of UTP14A in MGC803 and ECA109 cells with P16-KO, as determined by western blotting and RT-qPCR. (C) Effects of siUTP14A on the levels of phos-RB1 and total RB1 in MGC803 cells with and without P16-KO. (D) Comparison of FLAG-UTP14A expression efficiency in P16-WT and P16-KO MGC803 cells. (E) Comparison of the RB1 levels in P16-WT and P16-KO MGC803 cells with and without FLAG-UTP14A expression. (A and C) Three biological replicates (BioRep).

The protein but not the mRNA level of UTP14A was significantly lower in P16-KO cells than in P16-WT cells (Figures 5A and 5B), suggesting posttranslational regulation of UTP14A by P16. To evaluate the role of *UTP14A* downregulation in P16-KO-mediated degradation of RB1, we further analyzed the aforementioned siUTP14A cells and found that the RB1 level in siUTP14A P16-WT cells was as high as that in scrambled RNA control P16-KO cells, although the abundance of RB1 was increased in both P16-WT and P16-KO MGC803 cells (Figure 5C). Furthermore, we found that the amount of FLAG-UTP14A was much lower in P16-KO cells than in P16-WT cells (Figure 5D), suggesting that P16-KO increased UTP14A degradation. In the rescue experiment, the RB1 level in P16-KO cells with FLAG-UTP14A expression was fully restored to that in P16-WT cells without FLAG-UTP14A expression according to confocal microscopy analysis (Figure 5E), suggesting complete mitigation of P16-KO-mediated RB1 accumulation.

To study the mechanism of UTP14A downregulation by P16 loss, we blocked novel protein synthesis with CHX treatment in MGC803 cells and observed a significant decrease in the half-life of UTP14A in P16-KO cells relative to that in their P16-WT counterparts (Figure S4A). Furthermore, the difference in endogenous UTP14A levels was reversed in these cells when protein ubiquitination degradation was blocked with MG132 (Figure S4B). The difference in exogenous FLAG-UTP14A levels was also abolished after treatment with MG132 (Figure S4C). These results indicate that P16-KO may promote proteosome-related degradation of the UTP14A protein.

In addition, we also found that siUTP14A greatly increased the abundance of exogenous GFP-RB1 and its K143R mutant, whereas it only slightly increased that of its K810R mutant (Figure S5A). IP analysis also revealed that the level of ubiquitinated GFP-RB1 was markedly decreased by siUTP14A relative to that in scramble control cells, whereas the level of ubiquitinated RB1-K810R was greatly decreased in both scramble and siUTP14A cells, suggesting that the K810R mutation could block the ubiquitination of RB1, whether catalyzed by UTP14A or other E3 ligases (Figure S5B). Taken together, the aforementioned results revealed that UTP14A downregulation accounts for RB1 accumulation in cells with P16 loss.

### P16 induced the ubiquitination of RB1 in an S807/S811 phosphorylation-independent manner

The ubiquitination of some proteins (such as CCND1) may be dependent on protein phosphorylation.^34^ As we described previously, *UTP14A* downregulation induced by P16 loss not only increased the total RB1 level but also increased phos-RB1 (at S807 and S811). The phosphorylation sites S807 and S811 are two lysine residues surrounding the K810 ubiquitination site. It was suggested that the S807 and S811 sites might serve as priming sites to promote intermolecular interactions to facilitate further phosphorylation events^35^ and that the E3 ligase NRBE3 selectively binds hypophosphorylated RB1.^25^ Hence, we constructed two GFP-RB1 expression mutants with S811A and S807A&S811A replacements and used them to transfect MGC803 and ECA109 cells to study whether the phosphorylation of RB1 at S807 and S811 affects the P16-enhanced degradation of RB1. Western blot analysis revealed that the levels of both endogenous phos-RB1 and exogenous GFP-phos-RB1 S811A but not S807A&S811A mutant were increased by P16 KO in these cells, and the total endogenous RB1 and exogenous GFP-RB1 levels were also increased (Figure S6). The aforementioned data demonstrated that S807/S811-nonphosphorylated RB1 could be a P16-mediated ubiquitination target.

### P16-KO increased RB1 in a cell cycle progression-independent fashion

**Figure 6:**
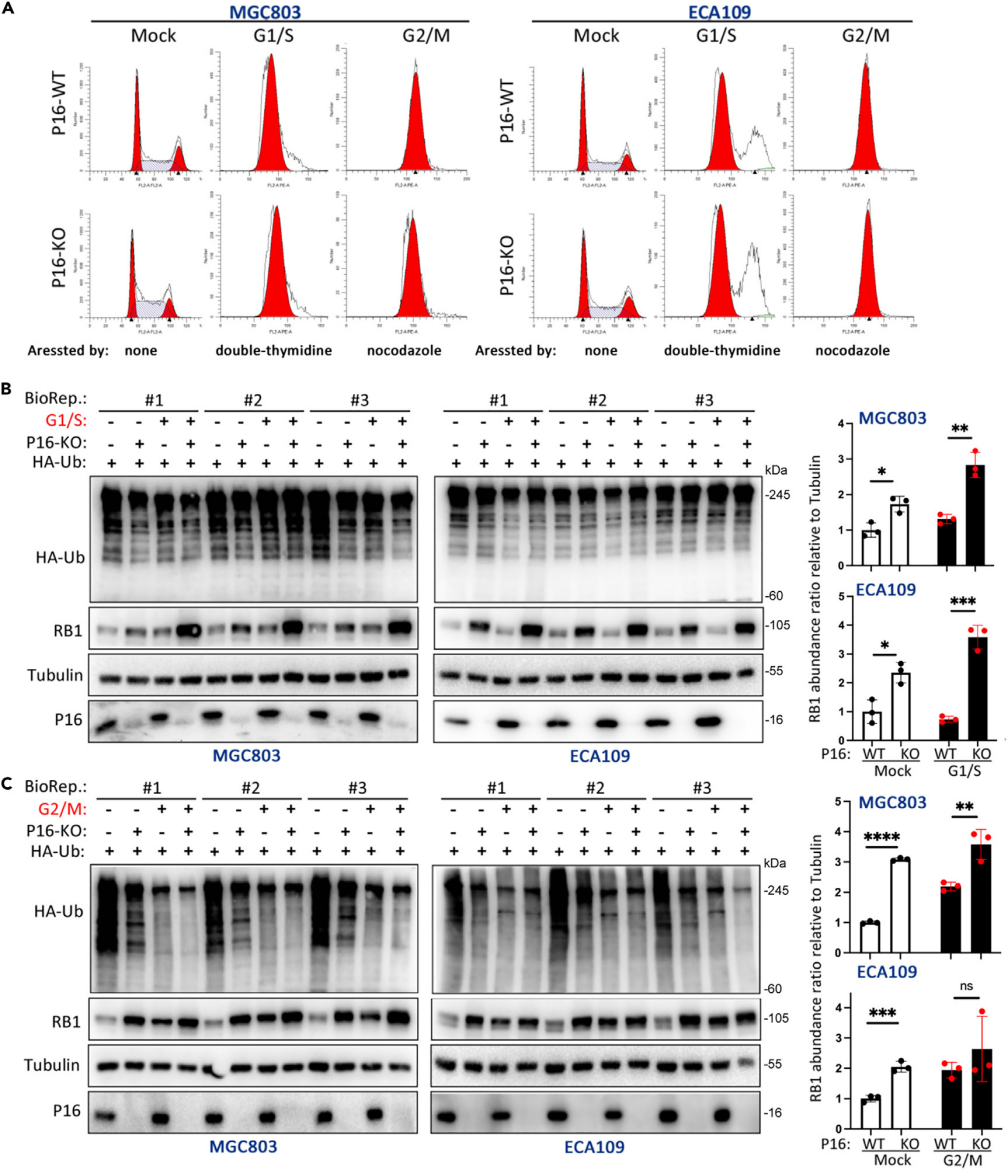
Comparison of RB1 accumulation and proteome-scale ubiquitination status in P16-WT and P16-KO cells arrested at various phases of the cell cycle (A) After treatment with double-thymidine or nocodazole, the cells were synchronized at the G1/S and G2/M phases via fluorescence-activated cell sorting (FACS). (B and C) The abundance of RB1 and total ubiquitinated proteins in cells synchronized at the G1/S and G2/M phases was determined via western blotting. The average RB1 protein abundance ratios relative to Tubulin are listed on the right for three biological replicates (BioRep).

Because RB1 inhibits the G1‒S transition^2^^,^^3^ and genetic or epigenetic *CDKN2A/P16* inactivation promotes cell proliferation,^36^^,^^37^^,^^38^ we studied whether P16-KO-mediated RB1 accumulation results from enhanced cell proliferation. We arrested cells at the G/S phase of the cell cycle by treatment with double-thymidine or at the G2/M phase by treatment with nocodazole (Figure 6A, right). The abundance of RB1 in P16-KO cells was still greater than that in P16-WT control cells after the cell cycle was arrested at these phases, especially in those cells arrested in the G1/S phase (Figures 6B and 6C). This phenomenon indicated that P16 decreases RB1 expression in a cell cycle progression-independent manner.

Unexpectedly, the level of total protein ubiquitination decreased greatly in P16-KO cells transfected with the HA-Ub vector, and restoring *P16* expression completely reversed the effect of P16-KO on total protein ubiquitination (Figure S7). When these cells were arrested at the G1/S or G2/M phase, the effect of P16-KO on total protein ubiquitination was abolished (Figures 6B and 6C). These results indicated that P16 loss also decreased the total protein ubiquitination level in a cell cycle progression-dependent manner.

### P16-KO increased cell proliferation in an RB1-dependent pattern

**Figure 7:**
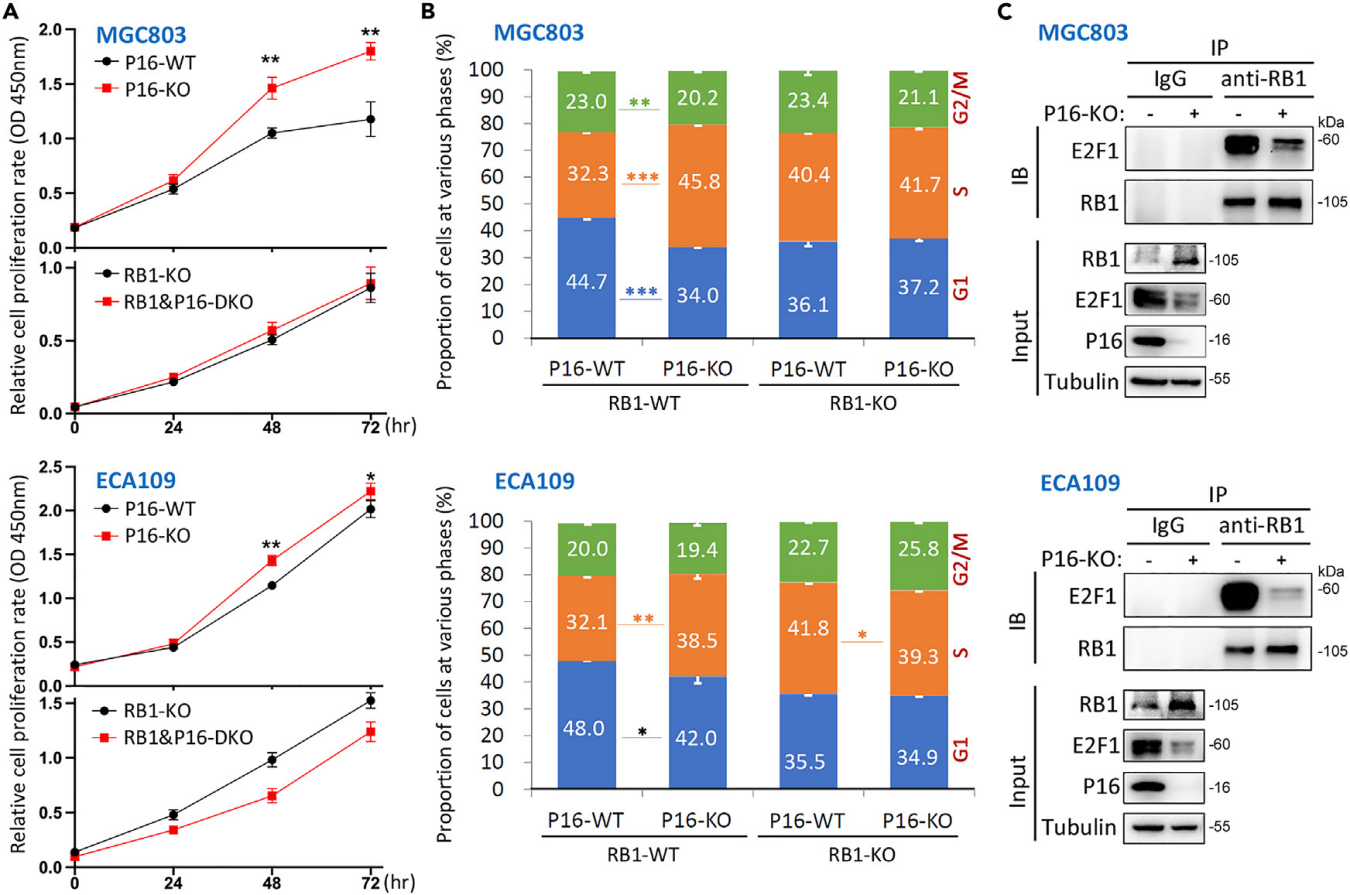
Effects of P16-KO-mediated RB1 accumulation on cell proliferation and E2F1 binding in human cells (A) Effects of P16-KO on the proliferation of cells with and without RB1-KO. (B) Proportions of cells with different *P16* and *RB1* genotypes at various cell phases. (C) The abundance of RB1 bound to E2F1 and target proteins in the input reference from cells with and without P16-KO in the coIP and western blot analyses. (A and B) Three biological replicates (BioRep).

To determine whether P16-mediated RB1 accumulation is functional, we initially performed cell proliferation and cell cycle analyses using RB1-KO cell models. We found that P16-KO significantly enhanced the proliferation of only RB1-WT cells but not RB1-KO cells (Figure 7A). Double KO of the *RB1* and *P16* genes (RB1 and P16-DKO) even inhibited the proliferation of ECA109 cells. Similarly, P16-KO consistently decreased the proportion of RB1-WT cells in the G1 phase but not that of RB1-KO cells (Figure 7B). These findings indicate that RB1 upregulation is essential for P16-KO-mediated increases in cell proliferation.

RB1 represses the activity of the transcription factor E2F1 on the promoters of cell cycle-related genes.^39^ We determined the difference in the abundance of E2F1 bound to RB1 between P16-WT and P16-KO cells by co-immunoprecipitation (coIP) analysis and found that the level of E2F1 bound to RB1 was greatly lower in P16-KO cells than in P16-WT cells (Figure 7C). Detailed analysis revealed that the abundance of E2F1 was greater in P16-WT cells than in KO cells. According to The Cancer Genome Atlas and GTEx RNA-seq datasets,^40^ *E2F1* expression is consistently correlated with *P16* but not *RB1* expression in human normal and cancer tissues (Figure S8). These results indicate that P16 loss induces *E2F1* downregulation and decreases RB1-E2F1 binding.

## Discussion

P16 inhibits the CDK4-catalyzed phosphorylation of RB1 and subsequently represses the proliferation of cells by regulating the G1–S transition in the cell cycle. Genetic and epigenetic inactivation of P16 promotes the development of many cancers.^36^^,^^37^^,^^38^^,^^39^^,^^41^^,^^42^^,^^43^^,^^44^^,^^45^ There is an inverse relationship between the expression of the *P16* and *RB1* genes in cancer cells. The transcription factor E2F1 may be involved in RB1-mediated control of *P16* gene expression.^46^ *P16* overexpression may upregulate *RB1* transcription.^9^ However, it is unknown whether loss of P16 function affects RB1 expression at the posttranslational level and contributes to the inverse expression relationship. Our current study demonstrates that the P16 protein drives the feedforward degradation of the RB1 protein through the ubiquitination of RB1 at K810 by UTP14A.

Consistent with previous reports,^9^ we also observed that the level of *RB1* mRNA increased in normal human fibroblast CCD-Co18 cells, in which *P16* was epigenetically inactivated by DNA methylation, as determined by RNA-seq and RT-PCR.^47^ However, we observed no significant difference in *RB1* mRNA levels between the tested cancer cell lines with and without gain or loss of *P16* function. Instead, we consistently observed a significant difference in the abundance of the RB1 protein between these cancer cell lines with and without gain or loss of *P16* function, indicating that there is a cell-specific posttranscriptional mechanism of P16 loss-mediated RB1 upregulation.

On the one hand, our findings and those of others indicate that there is a reciprocal negative regulatory feedback loop between the expression of the *RB1* and *CDKN2A/P16* genes. On the other hand, our work demonstrated that *CDKN2A/P16* is a functionally bidirectional gene. As a master tumor suppressor, P16 inhibits CDK4/6-catalyzed RB1 phosphorylation while simultaneously driving the feedforward degradation of RB1. When P16 is lost, the ubiquitination of RB1 at K810 by UTPA14 is inhibited, which consequently leads to RB1 accumulation in cells. This finding suggested that the *P16* gene may have previously unknown tumor suppressive potential and may play a role in mitigating the biological outcomes of *P16* inactivation by disrupting the negative feedback loop (i.e., inhibiting UTPA14 expression and K810 ubiquitination of RB1). In other words, there is a feedforward function for the WT *P16* gene, which only plays a role when it is inactivated.

The E3 ligase UTP14A selectively ubiquitinates RB1.^24^ However, the ubiquitination sites in RB1 induced by UTP14A have not been previously characterized. Here, we found that UTP14A ubiquitinated RB1 at K810 and that the K810R mutation abolished P16-enhanced RB1 ubiquitination and degradation. P16 increased the abundance of the UTP14A protein but not the mRNA level of the *UTP14A* gene, suggesting the posttranscriptional upregulation of UTP14A. The detailed mechanism of P16-mediated UTP14A upregulation needs to be studied further. UBA1 is an E1 upstream gene in the ubiquitin-proteasome system that indirectly degrades many proteins without target protein specificity. Our work revealed that RB1 levels were increased by *UBA1* knockdown in P16-KO cells but not in P16-WT cells. It is worth studying whether UBA1 may cooperate with UTP14A to ubiquitinate RB1 in P16-KO cells and contribute to a decrease in global protein ubiquitination.

K810 is also methylated by SET7/9, SMYD2, 53BP1, and PHF20L1.^21^^,^^22^^,^^23^^,^^24^ JMID3 demethylates RB1 at K810, which hinders the interaction of RB1 with CDK4 and decreases its phosphorylation at S807/S811.^34^ Both lysine and arginine residues are (mono/di/tri-)methylation sites, while lysine residues are ubiquitination sites within proteins by E3 ligases. Tri-methylated lysines cannot be ubiquitinated by E3 ligases due to the absence of a ubiquitin-binding nitrogen atom at the ε-amino group via tri-methylation. Theoretically, monomethylated or dimethylated lysine residues may be ubiquitinated by E3 ligases. For example, the E3 ligase WWP2 can specifically interact with K119-methylated Sox2 through its HECT domain to promote Sox2 ubiquitination,^48^ and PHF20L1 can bind to monomethylated DNMT1 at K142 and block its ubiquitination and degradation.^49^ It was also reported that NRBE3 selectively ubiquitinated the hypophosphorylated form of RB1.^25^ Although the replacement of K810 with R810 (K810R) completely prevented the impact of P16 on RB1 stability, it is not known whether the methylation status of RB1 at K810 may competitively affect K810 ubiquitination. The finding that abolishing S807/S811 phosphorylation with A807/A811 replacement did not affect RB1 K810 ubiquitination or degradation suggests that RB1 ubiquitination at K810 does not depend on phosphorylation at S807/S811 and that P16 drives the degradation of hypophosphorylated RB1 with biological functions.

To further determine whether the additional RB1 accumulation induced by *P16* deletion participates in the regulation of the cell cycle, we used RB1-KO cell models and found that the impact of *P16* deletion on cell proliferation depends on RB1 via cell cycle analysis.

RB1 not only represses the activity of the transcription factor E2F1^39^ but also promotes the transcription of cell differentiation genes through interactions with transcription factors such as MyoD, NF-IL6, and C/EBP, whereas it promotes SKP2 degradation in a cell cycle-independent fashion.^41^ These phenomena suggest that RB1 is a protein with multiple functions. According to our MS analysis, 76% of P16-KO-induced differential RB1-binding proteins (42 of 55) were nucleolar RNA-associated proteins (including UTP14A, NAT10, and UTP20/1A6). Consistent with other reports,^50^ we did not find interactions between RB1 and E2F in the MS analysis. However, RB1-E2F1 interactions were still detectable in P16-KO cells, even though the interactions were greatly decreased by P16-KO, probably due to the repression of *E2F1* expression by P16-KO. Although E2F1 is the known target of RB1, the genome-wide distribution of RB1 on chromatin has been technically challenging.^51^ Our MS analysis revealed that RB1 interacts mainly with nucleolar-associated RNA proteins (Data S2). Multiple nucleoli and enlarged nucleoli are markers of actively proliferating cells that increase protein synthesis. Studies on whether RB1 promotes rRNA biogenesis and cell proliferation through interactions with nucleolar-associated RNA proteins are warranted.

Both ubiquitination- and deubiquitination-related genes are therapeutic candidates. A ubiquitination-based tool called PROTAC has even been created to degrade many target proteins for the treatment of diseases.^52^ In the present study, we found an obvious decrease in total protein ubiquitination in cells with P16 loss in a cell cycle progression-dependent fashion, suggesting that intervening in global protein ubiquitination may be a novel strategy for the treatment of *P16*-deleted cancers.

In summary, our study demonstrated that *P16* and *RB1* reciprocally inhibit each other by forming a negative regulatory feedback loop. As a tumor suppressor, P16 simultaneously inhibits RB1 phosphorylation and destabilizes RB1 via UTP14a-catalyzed K810 ubiquitination. *P16* dysfunction leads to functional RB1 accumulation by interrupting the feedback loop. These findings provide insights into the role of the P16 protein in the regulation of RB1 function and may provide a strategy for the treatment of cancer patients with *P16* dysfunction.

### Limitations of the study

The mechanism of UTP14a degradation by the P16 protein has not been studied.

## Resource availability

### Lead contact

Further information and requests for resources should be directed to the lead contact, Dajun Deng (dengdajun@bjmu.edu.cn).

### Material availability

This study did not generate new unique reagents.

### Data and code availability

#### Data

This paper analyzes existing, publicly available data. The accessions for the datasets are listed in the key resources table. Original western blot images were deposited at Mendeley and are publicly available as of the date of publication. The DOI is listed in the key resources table. Additional information reported in this paper will be shared by the lead contact upon request.

#### Code

This paper does not report any original code.

#### Others

Any additional information required to reanalyze the data reported in this paper is available from the lead contact upon request.

## Acknowledgments

We appreciate Professor Xiaojuan Du, Basic Medical College at Peking University, for kindly providing the FLAG-hUTP14a expression vector. This work was supported by the 10.13039/501100001809National Natural Science Foundation of China (grant number 82372586), the Beijing Natural Science Foundation (grant number 7181002), and the Beijing Hospitals Authority' Mission Plan (code: SML20191101), China.

## Author contributions

W.W. conducted the experiments; B.Z. provided technical support; and D.D. designed the experiments, performed the bioinformatics analyses, received financial support, and wrote the paper. All the authors approved the manuscript.

## Declaration of interests

The authors declare no competing interests.

## STAR★Methods

### Key resources table

**Table:** 

REAGENT or RESOURCE	SOURCE	IDENTIFIER
**Antibodies**		

Rabbit monoclonal anti-p16INK4a	Abcam	Cat#ab108349; RRID:AB_10858268
Rabbit monoclonal anti-RB1	Abcam	Cat#ab181616; RRID:AB_2848193
Mouse monoclonal anti-GAPDH	Proteintech	Cat#60004-1-Ig; RRID:AB_2107436
Mouse monoclonal anti-Beta Tubulin	Proteintech	Cat#66240-1-Ig; RRID:AB_2881629
Mouse monoclonal anti-FLAG	Proteintech	Cat#66008-4-Ig; RRID:AB_2918475
Rabbit polyclonal anti-GFP	Proteintech	Cat#50430-2-AP; RRID:AB_11042881
Mouse polyclonal anti-HA	Proteintech	Cat#66006-2-Ig; RRID:AB_2881490
Rabbit polyclonal anti-UTP14A	Proteintech	Cat#11474-1-AP; RRID:AB_2272800
Mouse monoclonal anti-E2F1	Santa Cruz	Cat#SC-251; RRID:AB_627476
Rabbit monoclonal anti-Phospho-Rb (S807/811)	Cell Signaling Technology	Cat#8516; RRID:AB_11178658
Mouse monoclonal anti-Ubiquitin	Cell Signaling Technology	Cat#3936; RRID:AB_331292
Alexa Fluor 555-labeled Donkey Anti-Mouse IgG(H + L)	Beyotime	Cat#A0460; RRID:AB_2890133
Alexa Fluor 488-labeled Goat Anti-Rabbit IgG(H + L)	Beyotime	Cat#A0423; RRID:AB_2891323
Goat Anti-Rabbit IgG/HRP	Solarbio	Cat#SE134; RRID:AB_2797593
Goat Anti-Mouse IgG/HRP	Solarbio	Cat#SE131; RRID:AB_2797595

**Bacterial and virus strains**		

TransStbl3 Chemically Competent Cell	TransGen	Cat#CD521-01
Trans1-T1 Phage Resistant Chemically Competent Cell	TransGen	Cat#CD501-02

**Chemicals, peptides, and recombinant proteins**		

Cycloheximide (CHX)	Selleck Chemicals	Cat#S7418
MG132	Selleck Chemicals	Cat#S2619
Nocodazole	Beyotime	Cat#S1765
Puromycin Dihydrochloride	Beyotime	Cat#ST551
RIPA lysis buffer	Beyotime	Cat#P0013B
Thymidine	MCE	Cat#HY-N1150
SYBR Green PCR master mix reagents	Roche	Cat#4913914001
X-tremeGENE HP DNA Transfection Reagent	Roche	Cat#6366236001
Omifection-R Transfection Reagent	Omiget,	Cat#Omc-02
100× Protease Inhibitor Cocktail	Solarbio	Cat#IKM1010
Polyethylenimine Max	Polysciences	Cat#24765

**Critical commercial assays**		

Direct-Zol RNA MiniPrep Kit	Tianmo Sci & Tech Develop	Cat# R2050
First-Strand cDNA Synthesis Kit	TransGen	Cat#AT301-02
Protein A/G Immunoprecipitation Kit	Beaver	Cat#22202-100
Cell Counting Kit-8	Beyotime	Cat#C0043
Cell Cycle Assay Kit - PI/RNase Staining	Dojindo	Cat#C543

**Deposited data**		

Mass spectrometer (LC-MS)	This paper	Data S2
Original western blot images	This paper	https://doi.org/10.17632/6mp6cymc3c.1
Cancer Cell Line Encyclopedia, CCLE	Ghandi et al.^16^; Nusinow et al.^17^	https://sites.broadinstitute.org/ccle/
GEPIA website	Tang et al.^40^	http://gepia.cancer-pku.cn/
UbiBrowser 2.0	Wang et al.^29^	http://ubibrowser.bio-it.cn/ubibrowser_v3

**Experimental models: Cell lines**		

Human lung cancer cell line H1299	Laboratory of Chengchao Shou	N/A
Human lung cancer cell line A549	Laboratory of Zhiqian Zhang	N/A
Human gastric cancer cell line MGC803	Laboratory of Yang Ke	N/A
Human liver cancer cell line HepG2	Laboratory of Qingyun Zhang	N/A
Human breast cancer cell line MCF7	Laboratory of Yuntao Xie	N/A
Human embryonic kidney cell line HEK293FT	Laboratory of Yasuhito Yuasa	N/A
Human esophageal squamous cell line ECA109	Laboratory of Fen Liu	N/A
Human lung cancer cell line HCC827	National Laboratory Cell Resource Sharing Platform	N/A
Human lung cancer cell line H661	National Laboratory Cell Resource Sharing Platform	N/A
Human lung cancer cell line H358	National Laboratory Cell Resource Sharing Platform	N/A
Human lung cancer cell line H460	National Laboratory Cell Resource Sharing Platform	N/A
Human lung cancer cell line H1395	National Laboratory Cell Resource Sharing Platform	N/A
Human lung cancer cell line H292	National Laboratory Cell Resource Sharing Platform	N/A

**Oligonucleotides**		

Data S3 for Oligonucleotides	N/A	N/A

**Recombinant DNA**		

Plasmid: GFP-RB1	KHAN et al.^53^	Addgene Plasmid #16004
Plasmid: PX458	RAN et al.^54^	Addgene Plasmid #48138
Plasmid: FLAG-hUTP14a	LIU et al.^24^	N/A
Plasmid: FLAG-P16	Mailgene	Cat#MT00029
Plasmid: HA-ubiquitin (Ub)	Mailgene	Cat#MT00030
Plasmid: GFP-RB1 mutant plasmids (K143R, K810R, S811A, S807A&S811A)	Mailgene	N/A

**Software and algorithms**		

ImageJ software	Schneider et al.^55^	https://imagej.net/software/fiji/
GraphPad Prism 9.0 software	GraphPad	https://www.graphpad.com
Modifit software (Verity Software House, USA).	Verity Software House	N/A
ZEN Microscopy Software	ZEISS	N/A

### Experimental model and study participant details

#### Cell lines and cell culture

The human lung cancer cell lines H1299 and A549, the gastric cancer cell line MGC803, the liver cancer cell line HepG2, and the breast cancer cell line MCF7 were kindly provided by Professors Chengchao Shou, Zhiqian Zhang, Yang Ke, Qingyun Zhang, and Yuntao Xie at Peking University Cancer Hospital, respectively. HEK293FT cells were kindly provided by Professor Yasuhito Yuasa at Tokyo Medical and Dental University. The human esophageal squamous cell line ECA109 was kindly provided by Professor Fen Liu at Capital Medical University. These cell lines were tested and authenticated by Beijing JianLian Gene Technology Co. or Tianyi Huiyuan Biotech Co. before use. Short tandem repeat (STR) patterns were analyzed via the Goldeneye 20A STR Identifier PCR Amplification Kit. The human lung cancer cell lines HCC827, H661 H358, H460 H1395, and H292 were purchased from the National Laboratory Cell Resource Sharing Platform (Beijing, China) at the beginning of this study with STR authentication. The H1299, A549, MGC803, HepG2, H661, H358, and H460 cells were male, and the others were female. HEK293FT and MCF7 cells were cultured in DMEM (Gibco, Carlsbad, USA) supplemented with 10% fetal bovine serum (Gibco) and 1% penicillin‒streptomycin (Life Technologies, Carlsbad, USA), and the other cell lines were cultured in RPMI 1640 medium (Gibco). All the cells were maintained at 37°C in humidified air with 5% CO_2_ and placed in liquid nitrogen tanks for long-term preservation.

### Method details

#### Knockout of the CDKN2A/P16 and/or RB1 genes via CRISPR/Cas9

Single guide RNA (sgRNA) approaches^56^ were used to knock out the genetic sequence of the *CDKN2A/P16* and/or *RB1* genes. The sgRNAs individually designed for *CDKN2A* exon-1a (5′-accgt aacta ttcgg tgcgt tgg-3′) and RB1 exon-2 (5′-caccg agaga gagct tggtt aact-3′) were created over an online platform specifically available at a website (http://crispr.mit.edu). These sgRNAs were cloned and inserted into the PX458 vector (Plasmid #48138, Addgene, USA) and transfected into MGC803 and ECA109 cells. Then, a flow-sorting assay was performed for green fluorescence with a FACSCalibur flow cytometer (BD Biosciences, Franklin Lakes, USA) 48 h posttransfection. Two weeks later, monoclonal cells in good growth conditions were selected, and the KO status of the target sequence in the subclones was identified by DNA sequencing (Figure S2) and Western blotting.

#### Cell-cycle arrest and flow cytometry analysis

##### Cell-cycle arrest

First, the cells were inoculated in a 6-well plate at the appropriate density to prevent contact inhibition, and the experiment continued after the cells were attached. To arrest the cells in the G1/S phase of the cell cycle, MGC803 and ECA109 cells were treated with thymidine at final concentrations of 1.0 and 2.0 mmol/L, respectively, for 16 h, after which the cells were released for 9 h, after which the protein lysate was collected after the second round of treatment with thymidine for 16 h.^57^ To arrest cells at the G2/M phase, MGC803 and ECA109 cells were treated with nocodazole at concentrations of 50 and 100 ng/mL for 12 h.^58^

##### Cell cycle analysis

Exponentially growing cells were trypsinized and collected via centrifugation. After being washed with PBS, the cells were resuspended in 70% ice-cold ethanol and incubated at 4°C overnight. In accordance with the instructions of the Cell Cycle Assay Kit (Dojindo, Kumamoto, Japan), the cells were incubated with RNase and propidium iodide at 37°C for 30 min and analyzed with a BD Accuri C6 flow cytometer (BD Biosciences) and Modifit software.

##### Western blotting

The cells were collected and lysed at approximately 80% confluence. Proteins were separated by SDS‒PAGE at different concentrations according to the molecular weight of the target protein and transferred onto a PVDF membrane. The membrane was subsequently blocked with 5% fat-free milk overnight at 4°C and incubated with primary antibodies against P16 (1:3000, Abcam, UK), anti-RB1 (1:2500, Abcam, UK), phospho-RB1 (Ser807/811) (1:2500, Cell Signaling Technology, USA), GAPDH (1:10000, Proteintech, USA), E2F1 (1:1000, Santa Cruz, USA), and UTP14A (1:500, Proteintech, USA) for 1 h at room temperature. After three rounds of washing with PBST (PBS with 0.1% Tween 20), the membrane was further incubated with goat anti-rabbit (1:2000, Solarbio, China) or goat anti-mouse (1:2000, Solarbio, China) secondary antibodies at room temperature for 1 h. After washing 3 times, protein signals were visualized via the Immobilon Western Chemiluminescent HRP Substrate Kit (WBKLS0500, Millipore, Billerica, USA).

##### RNA extraction and qRT‒PCR

Total RNA was extracted from cells at approximately 70% confluence via a Direct-Zol RNA MiniPrep Kit (Tianmo Sci & Tech Develop, Beijing, China). The quality and concentration of the RNA samples were monitored via a NanoDrop 2000 system (Thermo Fisher Scientific, Waltham, USA). Qualified RNA samples were reverse transcribed via a First-Strand cDNA Synthesis Kit (TransGen Biotech, Beijing, China). SYBR Green PCR master mix reagents (Roche, Mannheim, Germany) were used to perform qRT‒PCR with an Applied Biosystems 7500 Real-Time PCR device (Thermo Fisher Scientific, Waltham, USA). The relative expression levels of the tested genes were normalized to those of Alu RNA references via the classical ΔΔCt method.^59^ The sequences of the primers used in these PCR assays are listed (Data S3) and were synthesized by Tianyi Huiyuan Biotech Co.

##### Plasmid transfection

Transient transfection was performed with X-tremeGENE HP DNA Transfection Reagent according to the manufacturer’s instructions (Roche, Mannheim, Germany). Plasmids, including transgenes and packaging plasmids, were cotransfected into HEK293FT cells via Polyethylenimine Max (Polysciences, Connecticut, USA) according to the manufacturer’s protocol for stable transfection. The viruses were collected after 48 h. When the tumor cells reached a density of approximately 60–70%, they were infected with the collected viruses for 48 h. Stably infected cells were selected for 5 days with 1 μg/mL puromycin (Beyotime Biotech, Shanghai, China). Transfection efficiency was monitored by Western blotting.

##### siRNA-mediated knockdown of gene expression

Two synthesized duplex small interfering RNA oligos (siRNAs) were synthesized for each target gene from Mailgene (Beijing, China) and are listed in Data S3. Cells at 70–80% confluence were transfected with a gene-specific siRNA pair or scramble control RNA via Omifection-R Transfection Reagent (Omiget, Beijing, China) according to the manufacturer’s manual, and the media was changed 6–12 h after transfection. The knockdown efficiency was determined via Western blotting or quantitative RT‒PCR (qRT‒PCR).

##### Protein immunoprecipitation (IP)

Cell lysates were prepared in RIPA buffer with 1 × protease inhibitor cocktail (Solarbio, Beijing, China) and used directly for immunoprecipitation with a BeaverBeads Protein A/G Immunoprecipitation Kit (Beaver Biotech, Suzhou, China) according to the manufacturer’s instructions. First, 3 μg of the appropriate antibodies against RB1, GFP, or rabbit IgG were coupled with protein A/G magnetic beads for 2 h at 4°C and then incubated with the cellular extracts for 12 h at 4°C on a rotating platform. After extensive washing with wash buffer (approximately 6 times), the beads were resuspended in 40 μL of 1×SDS loading buffer and boiled for 5 min at 95°C to obtain precipitated proteins for subsequent analysis.

##### RB1 ubiquitination analysis

The cells were transfected with HA-ubiquitin, WT-GFP-RB1, or mutant-GFP-RB1 vectors for 48 hrs. The cell lysate was prepared by the addition of 1% SDS and boiling at 95°C for 10 min to disrupt the interaction and retain the covalent modification for the ubiquitination analysis. The cell lysate was incubated with 3 μg of anti-RB1, anti-GFP or rabbit IgG antibodies for 12 h at 4°C. RB1 ubiquitination was analyzed by immunoblotting with an anti-HA antibody (1:1000, Proteintech, USA) or an anti-Ub antibody (1:1000, Cell Signaling Technology, USA). The detailed immunoprecipitation procedures are described previously.^60^

##### Mass spectrometer (LC‒MS)

RB1 antibody was added to MGC803 P16-WT and P16-KO cell lysates for IP. Rabbit IgG was used as a negative control. All the precipitated proteins obtained in the IP experiment were separated on SDS‒PAGE gels for approximately 10 min and stained with Coomassie blue. After decolorization, the SDS‒PAGE gel was preserved in water, and liquid chromatography connected to a mass spectrometer (LC‒MS) was performed at the National Center for Protein Sciences at Peking University in Beijing, China (Data S2).

##### Confocal immunofluorescence assay

The cells grown on glass cover slips were fixed and permeabilized with ice-cold ethanol (100%) for 10 min at 0°C and blocked for 60 min with 5% BSA at room temperature. Then, the cells were incubated with primary antibodies [anti-RB1 (1:200, Abcam, UK) or anti-FLAG (1:400, Protein Tech, USA)] for 1 h at room temperature and secondary antibodies [Alexa Fluor 488-conjugated goat anti-rabbit IgG (H + L) (1:200, Beyotime, Shanghai, China) or Alexa Fluor 555-conjugated donkey anti-mouse IgG (H + L) (1:200, Beyotime, Shanghai, China)] for 1 h at room temperature. Nuclei were counterstained with DAPI. The cells were visualized, and images were obtained with a Zeiss confocal microscope (Oberkochen, Germany).

##### Cell viability assay

The proliferation abilities of these cells were determined with a Cell Counting Kit-8 (Beyotime Biotech, Shanghai, China) following the product specifications. In brief, cells were seeded into 96-well plates at a density of 2.5–3.5 ×10^3^ cells per well according to the cell growth rate, and 10 μL of CCK-8 reagent was added to each well every 24 h. Finally, the absorbance at a wavelength of 450 nm was measured via a SpectraMax iD3 microplate reader (Molecular Devices, Shanghai, China).

### Quantification and statistical analysis

GraphPad Prism 9.0 software was used to perform the statistical analysis. The Kolmogorov-Smirnov test or Shapiro‒Wilk test was used to test whether the data conformed to a normal distribution. Spearman correlation analysis was used to assess the relationship between the mRNA and protein abundances of the *CDKN2A* and *RB1* genes. Two-tailed unpaired Student’s t tests or nonparametric Mann‒Whitney U tests were used to compare differences between two groups. In Figure S1A, n represents the number of cell lines; in Figures 1B, 5D, 5E, and S5C, n represents the number of cells counted; and n in the other figures represents the number of biological replicates. The data are presented as the mean with the standard deviation (SD). *p* < 0.05 (two-sided) was considered to indicate a statistically significant difference. Significance was set at *p* < 0.05 (∗), <0.01 (∗∗), <0.001 (∗∗∗), or <0.0001 (∗∗∗∗).

### Additional resources

This paper has not generated or contributed to a new website/forum, and it is not part of a clinical trial.

## Supplemental information

Document S1. Figures S1–S8

Data S1. Lists of known and predicted RB1-related ubiquitinases and deubiquitinases

Data S2. List of the differential RB1-binding proteins between MGC803 cells with and without P16-KO, as determined by LC-MS analysis

Data S3. Sequences of primers and interference RNAs
