# Supplementary material for: P16INK4A drives RB1 degradation by UTP14A-catalyzed K810 ubiquitination
Source: iScience. 2024 Sep 3;27(10):110882. doi: 10.1016/j.isci.2024.110882 (PMC11440251; doi:10.1016/j.isci.2024.110882)
Supplement: Document S1. Figures S1–S8 [file mmc1.pdf]

iScience, Volume *27*

Supplemental information

$P16^{INK4A}$ drives RB1 degradation by UTP14A-catalyzed K810 ubiquitination

Wenjie Weng, Baozhen Zhang, and Dajun Deng

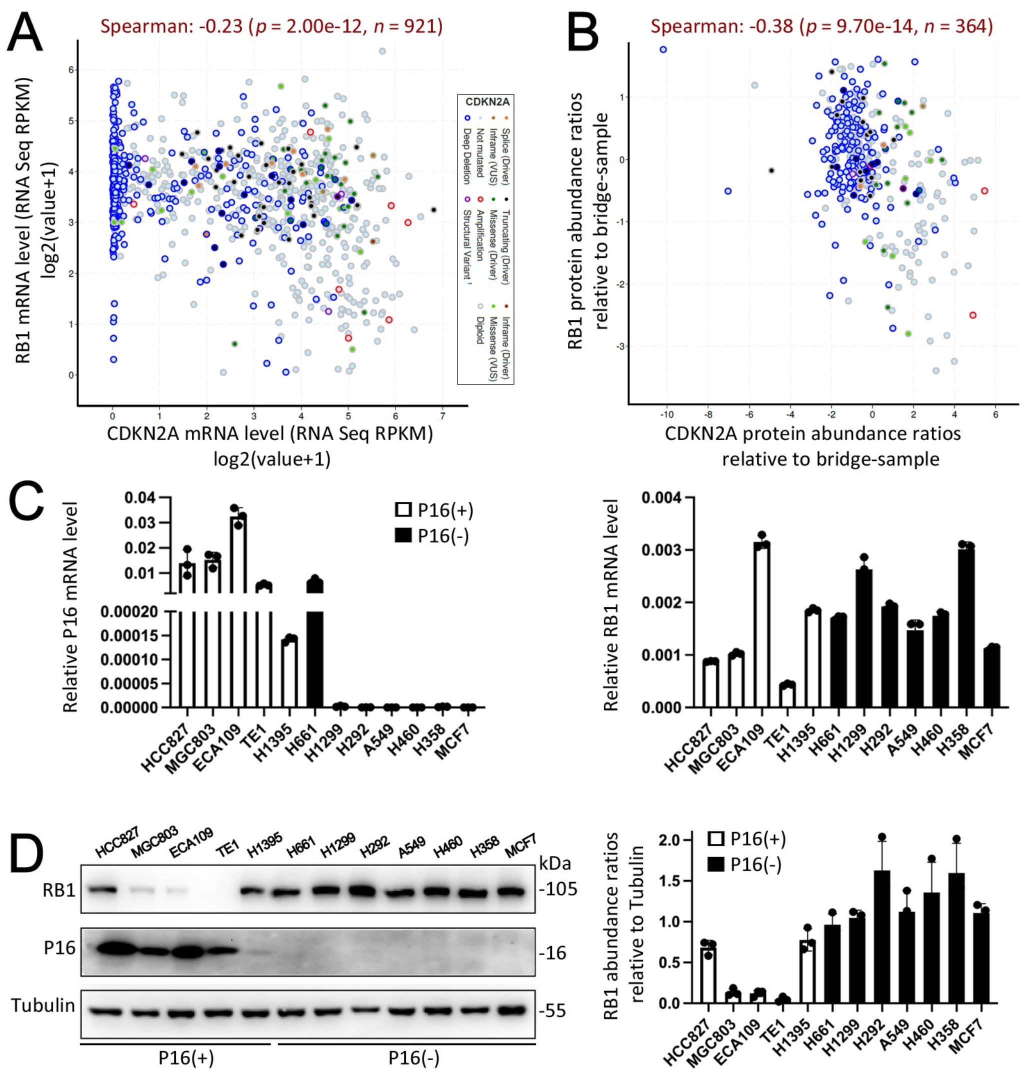

Figure S1. Association between the expression levels of the CDKN2A/P16 and RB1 genes. (**A** and **B**) The reverse relationship between the mRNA and protein abundances of the *CDKN2A* and *RB1* genes in cancer cell lines in the CCLE datasets[16,17]. The correlation efficiency and p value in the Spearman correlation test are labeled. (**C** and **D**) The mRNA and protein abundances of the *CDKN2A* and *RB1* genes in 12 human cancer cell lines. The band density for RB1 was calculated via ImageJ software, and the average ratio of the relative abundance of RB1 to that of Tubulin is presented as the mean with the standard deviation (SD) for three technical replicates.

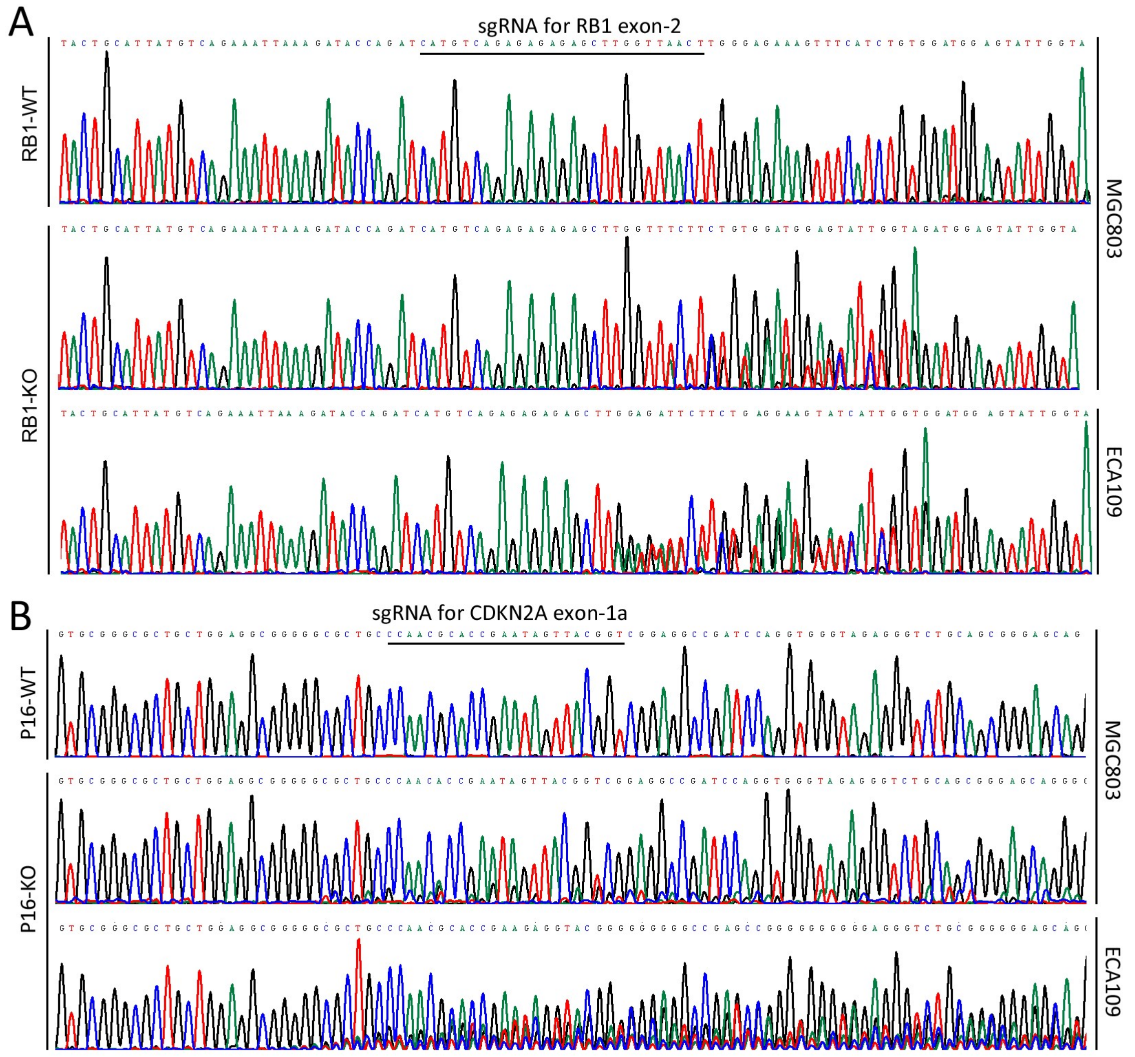

Figure S2. The results of DNA clone sequencing were used to determine the status of target gene knockout (KO) in MGC803 and ECA109 cells via CRISPR/Cas9. (**A**) The genetic sequences of *RB1* exon 2 in pooled subclones with and without *RB1*-specific single guide RNA (sgRNA) transfection. (**B**) The genetic sequences of *CDKN2A* exon-1a in pooled subclones with and without *CDKN2A*-specific sgRNA transfection.

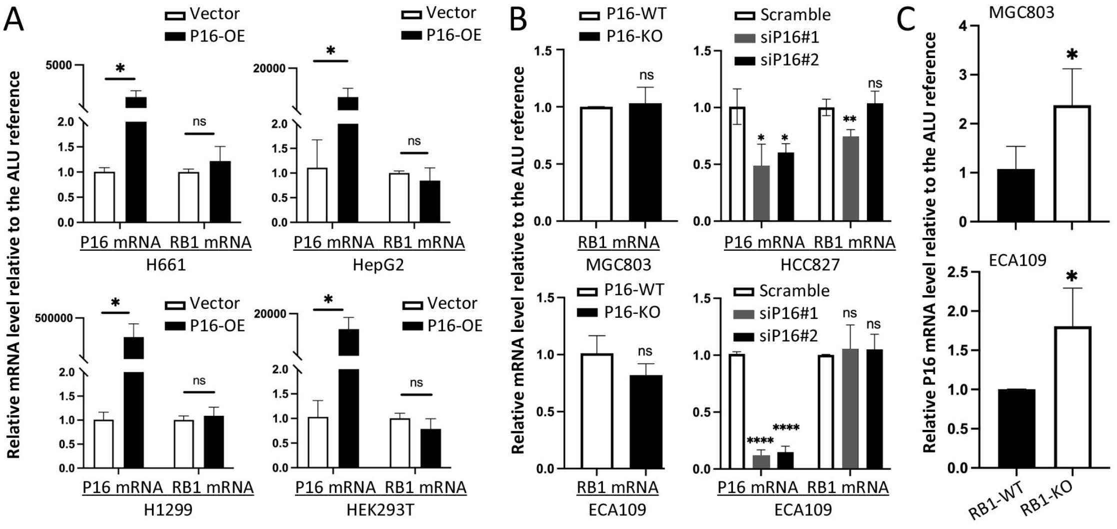

Figure S3. Effect of functional changes in the *CDKN2A/P16* gene on *RB1* mRNA levels in cancer cell lines and vice versa. (**A** and **B**) Impact of P16-OE, P16-KO, and siRNA-mediated knockdown of *P16* on the mRNA levels of the *RB1* gene in different cancer cell lines, as determined by qRT-PCR. (**C**) Impact of *RB1* KO on *P16* mRNA levels in the MGC803 and ECA109 cancer cell lines. */**/****: $p < 0.05/0.01/0.0001$ according to unpaired Student's t test.

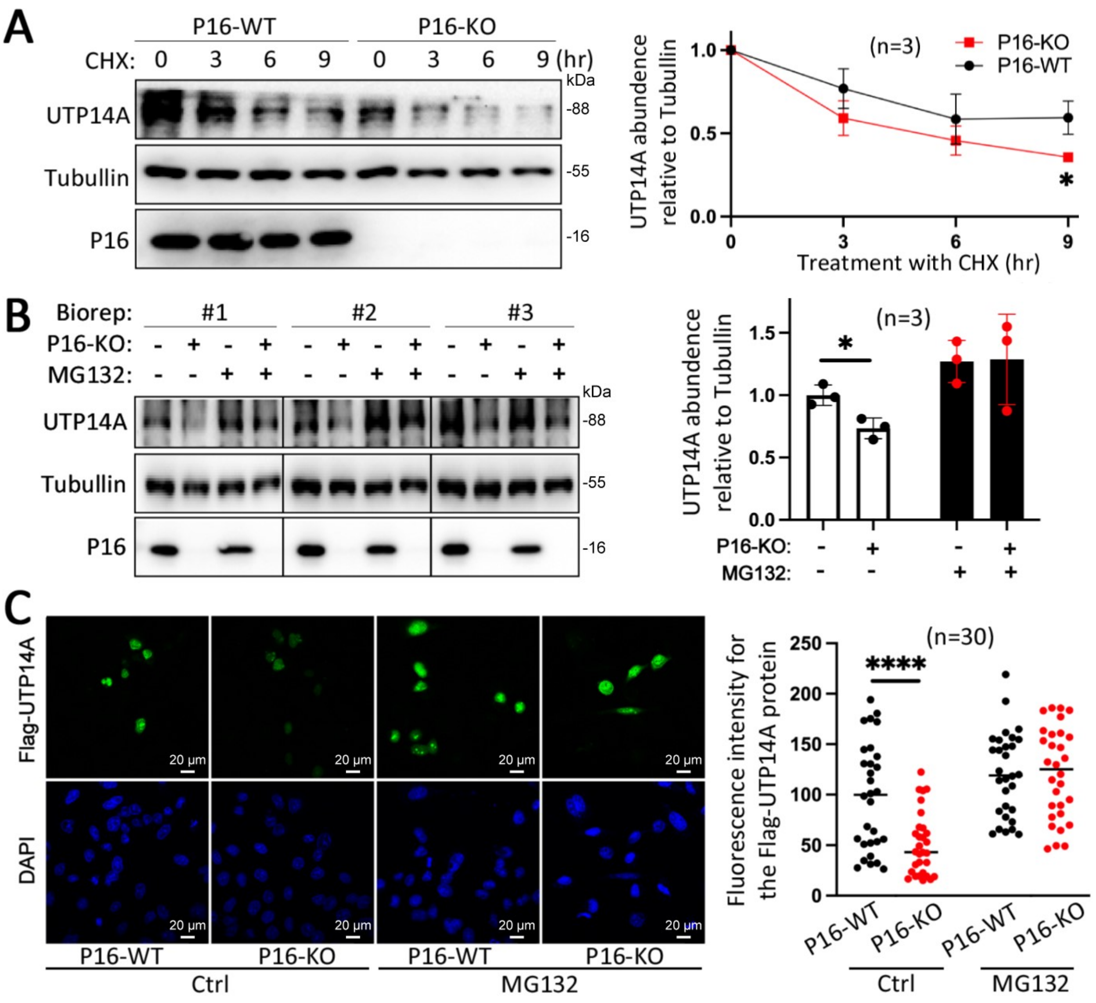

Figure S4. Effect of CHX and MG132 treatments on P16-mediated UTP14A regulation in MGC803 cells. (**A**) Effect of CHX treatment (final concentration, 50 μg/mL) on endogenous UTP14A stability in P16-WT and P16-KO cells at various time points, as determined by Western blotting. The average UTP14A abundance in three biological replicates at each time point is displayed on the right. (**B**) Effects of MG132 treatment (25 μM) for 4 hrs on endogenous UTP14A stability in P16-WT and P16-KO cells. (**C**) Effects of MG132 treatment on exogenous Flag-UTP14A stability in P16-WT and P16-KO cells. */****: p< 0.05/0.0001 according to unpaired Student's t test, respectively.

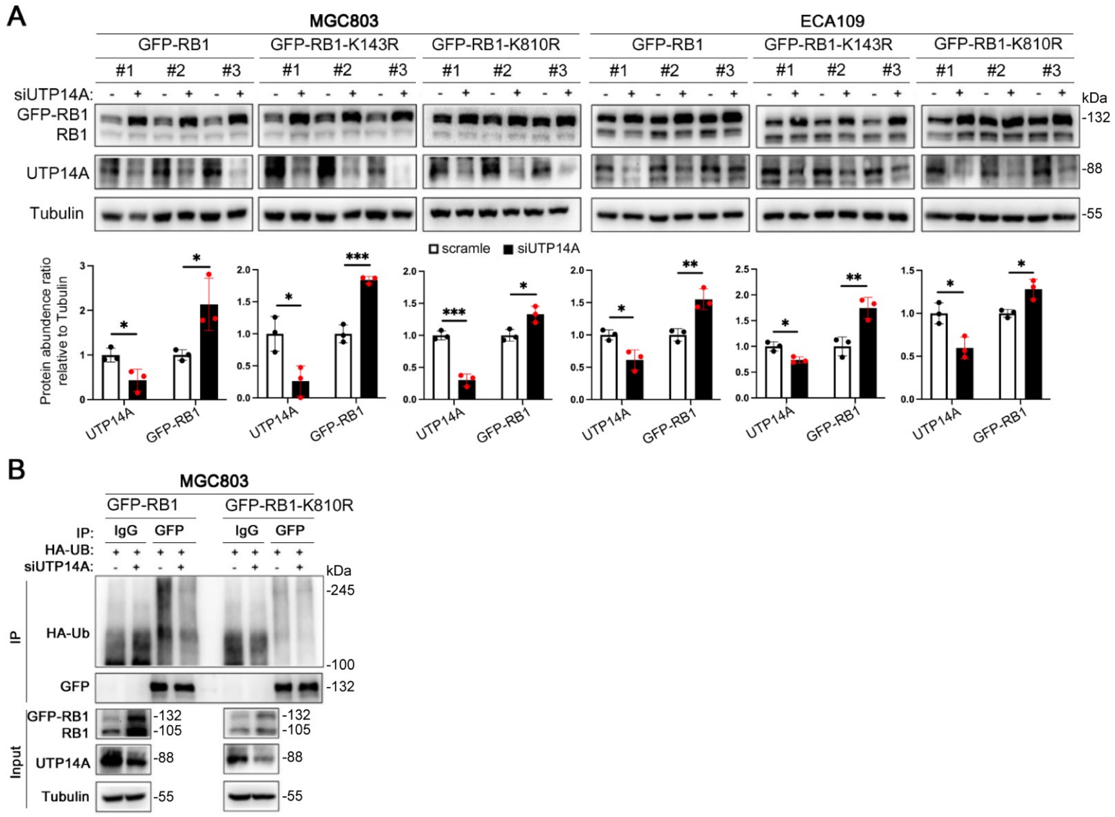

Figure S5. Effect of *UTP14A* knockdown on RB1 degradation and ubiquitination. (**A**) Impact of siUTP14A on the abundance of GFP-RB1 proteins with and without deletion of ubiquitination sites in MGC803 and ECA109 cells. (**B**) Impact of siUTP14A on the ubiquitination levels of GFP-RB1 proteins with and without deletion of ubiquitination sites in MGC803 cells. */**/***: p < 0.05/0.01/0.001 according to unpaired Student's t test.

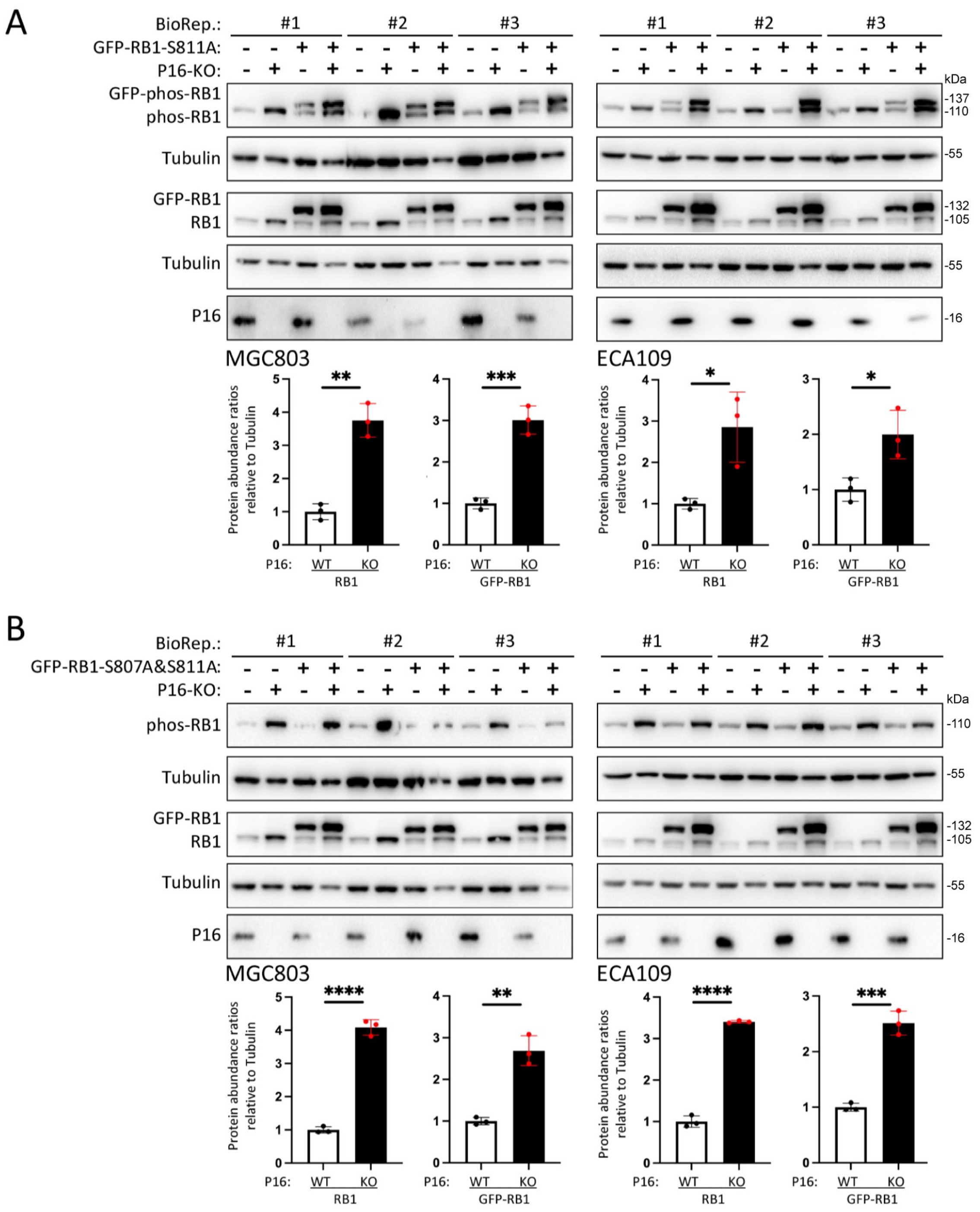

Figure S6. Effect of abolishing the phosphorylation of S807 and S811 on RB1 degradation induced by P16. (**A** and **B**) Results for the RB1-S807A and RB1-S807A&S811A mutants. */**/***/****: $p < 0.05/0.01/0.001/0.0001$ according to unpaired Student's t test.

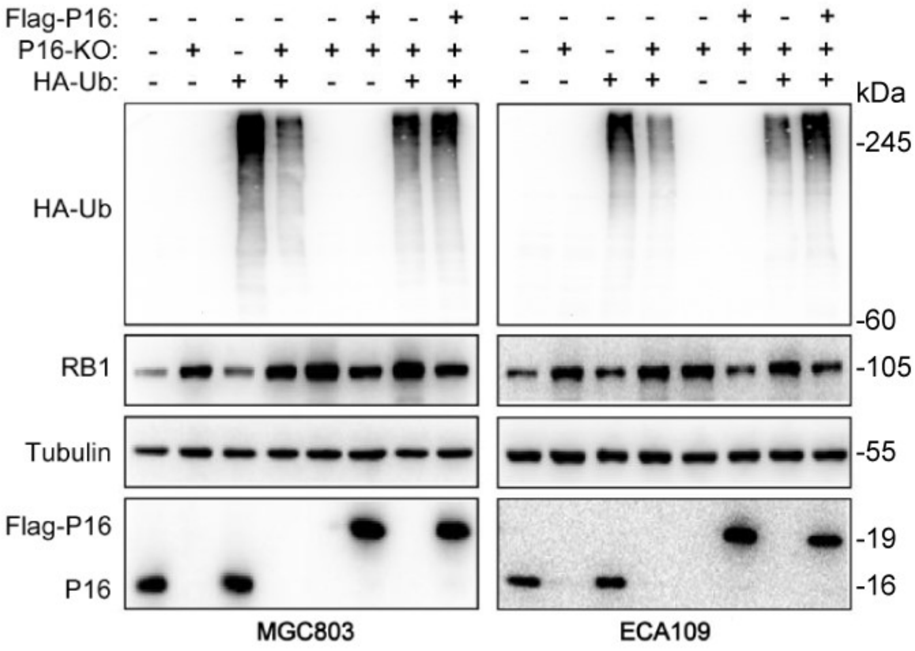

Figure S7. Effects of P16-KO on the level of whole proteome-wide ubiquitination in MGC803 and ECA109 cells

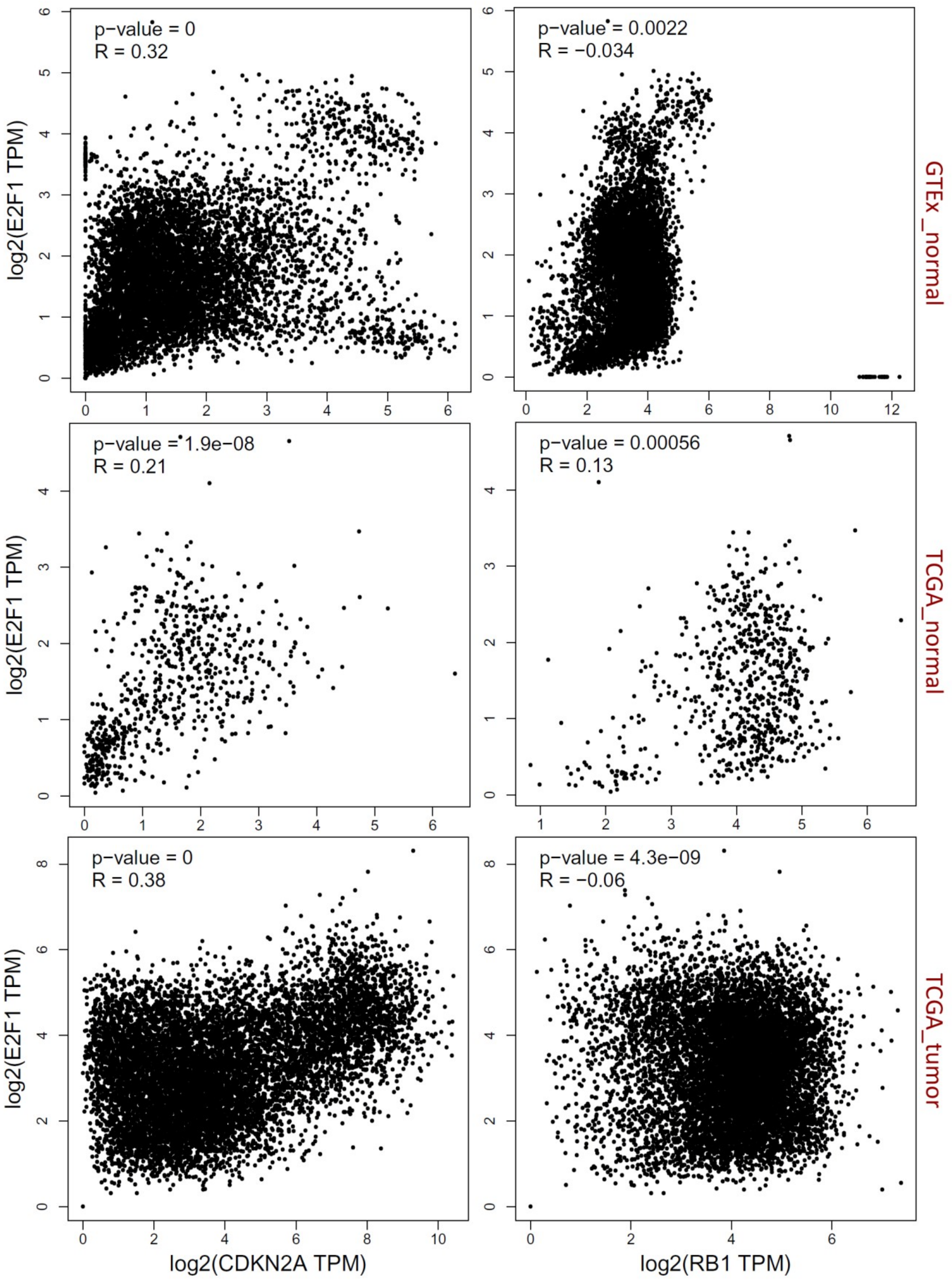

Figure S8. Correlations between the expression levels of the *CDKN2A/P16* and *RB1* genes in normal human tissues in the GTEx project and normal and carcinoma tissues in the TCGA project. These charts were adapted with images downloaded from the GEPIA website (http://gepia.cancer-pku.cn/)[40].
